# The Influence of Intrapersonal, Interpersonal, and Community Protective Factors on Hong Kong Adolescents’ Stress Arising from Political Life Events and Their Mental Health

**DOI:** 10.3390/ijerph18189426

**Published:** 2021-09-07

**Authors:** Ching-Wen Chang, Siu-Ming To, Wallace Chi-Ho Chan, Alex Ching-Pong Fong

**Affiliations:** 1 Graduate Institute of Social Work, National Taiwan Normal University, Taipei 106, Taiwan; cwchangswk@ntnu.edu.tw; 2 Department of Social Work, The Chinese University of Hong Kong, Hong Kong, China; chchan@swk.cuhk.edu.hk (W.C.-H.C.); alexfong@cuhk.edu.hk (A.C.-P.F.)

**Keywords:** youth mental health, stress arising from political life events, intrapersonal protective factors, interpersonal protective factors, community protective factors

## Abstract

Precarious political circumstances can take a mental toll on young people. Adopting a socio-ecological perspective, this study investigated the influence of stress arising from political life events, intrapersonal factors (i.e., meaning in life, resilience), interpersonal factors (i.e., social support, associational social capital), and community factors (i.e., perceived empowerment in the community, perceived opportunities for civic engagement) on the mental health of youth in Hong Kong. Furthermore, it examined the moderating effects of these factors on the relationship between stress arising from political life events and mental health. A cross-sectional quantitative survey with a stratified purposive sampling data collection method was conducted. A total of 1330 secondary school students were recruited for this study. Multiple regression analysis was performed to examine both direct and moderation effects. The results indicate that high stress arising from political life events, low meaningfulness in life, low resilience, low social support, low youth empowerment in the community, and high civic engagement in the community were related to high mental distress. None of the presumed moderators moderated the relationship between stress due to political life events and mental distress. Assessing and addressing stress due to political life events would be potentially important in mental health programs for Hong Kong adolescents in precarious political situations.

## 1. Introduction

Precarious political situations can manifest in the forms of uncertain political futures, political conflicts, political or religious extremism, terrorism, or humanitarian crises. Such forms of socio-political instability can take a mental toll on young people who are already dealing with the identity and existential issues that commonly occur during adolescence [1,2]. When youth fail to find meaning in their lives or struggle to cope with difficulties amid precarious socio-political situations, they may be confronted with an existential crisis or a sense of powerlessness, which are considered to be etiological factors of psychopathology [3]. Given the prevalence and unexpected nature of such circumstances [4], there is an urgent need to conduct research to examine the wellbeing of young people amid precarious political situations, and to investigate the socio-political, community, interpersonal, and intrapersonal factors that may influence youth’s mental health during major political life events.

### 1.1. Mental Health in the Recent Social Movement in Hong Kong

According to The World Health Organization (WHO), mental health can be understood as “a state of well-being in which the individual realizes his or her own abilities, can cope with the normal stresses of life, can work productively and fruitfully, and is able to make a contribution to his or her community” [5]. The 2014 Umbrella Movement in Hong Kong is an information-rich case for the impact of mass socio-political movements on population mental health. After the Umbrella Movement, there were high percentages of moderate to severe cases of anxiety (59.69%) and depression (22.48%) among Hong Kong people between 18 to 24 years of age [6]. A longitudinal study showed that Hong Kong citizens who were older, less educated, unemployed, and making a lower household income were more likely to demonstrate persistent moderate depression after the Umbrella Movement. Interestingly, this study also found that those between 15 to 34 years of age demonstrated the lowest instances of persistent moderate depression (25.5%) [7].

Two other studies in Hong Kong closely examined how participation in the Umbrella Movement affected a person’s wellbeing. The first study, conducted by Lau et al. [8], found that the three types of responses to the Umbrella Movement (i.e., worries about safety, negative emotional responses to media reports, and conflicts with peers about the Movement) and the emotional responses to local political situations were both significantly associated with mental distress. In contrast, personal participation in the movement was not associated with any mental distress. The other study found that social resource loss on social media (e.g., losing friends or followers on social media) was associated with depressive symptoms among those between 39 years of age and older. However, this did not hold for younger persons between 18 to 38 years of age [6].

Although previous studies demonstrated the importance of examining the psychosocial responses to political movements, they focused heavily on a psychosocial perspective that positions political tension as a structural factor of general public mental health [9]. This means that political conflicts are largely viewed as socially produced results that can negatively impact population mental health. There is, however, little consideration for how precarious socio-political contexts affect the overall functioning of youth and the specific, nuanced aspects of a young person’s lived experiences. Consequently, a purely psychosocial approach may overlook the variety of conflicts encountered by youth, ignore contextual factors, and ultimately neglect how young people’s lived experiences in socio-political events may affect their mental wellbeing. In addition, these existing studies were not focused on adolescents. Because adolescents are currently increasingly active participants of socio-political actions [10], there is a great need to examine how youth are exposed to and affected by contemporary socio-political situations. The 2019 Anti-Extradition Law Amendment Bill (“ELAB”) Incident and resultant protests in Hong Kong spawned ongoing socio-political unrest that directly and indirectly affected the livelihood and well-being of locals [11,12,13]. This incident was caused by the Hong Kong Special Administrative Region (HKSAR) Government’s proposed amendment of the existing Fugitive Offenders Ordinance and Mutual Legal Assistance in Criminal Matters Ordinance that would have allowed people in Hong Kong to be extradited to Mainland China. As a result, massive demonstrations, protests, and serious confrontations between protesters and the police occurred in different districts of Hong Kong. After the announcement of the HKSAR government’s formal withdrawal of the Anti-Extradition Law Amendment Bill, the promulgation of the national security law, as well as the arrest of active politicians and protest leaders by the authorities under the national security law, the ELAB Incident, and its related protests, came to an end. Nevertheless, how the socio-political tension after the ELAB Incident affected adolescents’ mental wellbeing remains largely unknown. Since today’s youth hold higher expectations of their political, civil, and social rights, the current socio-political environment has undoubtedly brought about massive internal and external changes for young people in Hong Kong, all of which can severely impact mental health.

### 1.2. The Effect of Stress Arising from Political Life Events on Youth Mental Health and Wellbeing

Based on the notion that a person’s perceptions of and reactions to stressful events are highly transactional and dynamic, Slone [14] argued that “adverse political life events” represent a unique category of life stressors because they are located within a particular historical and political context and thus can impact collective experiences and behaviors.

A comparative study on Israeli children and Palestinian children aged 12 to 13 found that, while Palestinian children reported greater exposure to political violence, the severity of such exposure led to reduced mental distress among this group [15]. Her findings show that 98 children and youth may experience the surrounding socio-political events differently depending on their subjective appraisal.

### 1.3. Adopting a Socio-Ecological Perspective in Youth Mental Health 

Traditional psychological models of youth mental health are criticized for their sole focus on a person’s psychological problems, without much regard for contextual and social factors (e.g., [16]). Alternatively, a socio-ecological perspective considers youth and their surrounding environments as a unitary system, with particular attention on the relationships between the specific and unique social, cultural, and political contexts [4]. This perspective is premised on two core ideas. First, researchers should recognize youth and their surrounding social systems, and any reciprocal relationships between them. Second, researchers should use different levels of analysis and examine the transactions between risks and protective factors at different socio-ecological levels, such as family, peer, school, community, and other social levels [16]. By adopting a socio-ecological perspective, a conceptual framework can be established for investigating the individual-environmental transactions that affect adolescent well-being amid socio-political precarity [2]. Specifically, this framework can be used to examine the direct and moderating effects of different intrapersonal, interpersonal, and community protective factors on the relationship between adolescents’ stress due to political life events and their mental health.

### 1.4. The Influence of Intrapersonal Protective Factors on Youth Mental Health and Wellbeing in Political Conflicts

Intrapersonal protective factors generally refer to an individual’s inner perceptions or characteristics. Naturally, meaning making may have a particularly important role for youth who are dealing with highly anxious provoking and stressful experiences induced by precarious socio-political contexts [1,17,18]. Because one’s construction of meaning operates at both personal and interpersonal levels and can be heavily influenced by his or her values and beliefs regarding current social or political events, exploring how a youth’s mental health is influenced by his or her perceived meaning in life can greatly enhance our understanding of youth functioning, engagement, and coping during and after socio-political conflicts.

Resilience can be defined as the capacity, processes, or outcomes of successful adaptation in the context of significant threats to function or development [19]. Individuals with a higher level of resilience tend to possess the following: (1) belief in their competence to cope with stressful and challenging events; (2) the ability to deeply engage in or to commit themselves to their life tasks; and (3) the ability to view crises as opportunities for further development [20]. These characteristics are often associated with how individuals perceive and respond to potential stressors in severe political life events [21,22].

### 1.5. The Influence of Interpersonal and Community Protective Factors on Youth Mental Health and Wellbeing in Political Conflicts

Generally speaking, interpersonal and community protective factors consider an individual’s functioning within a social environment, and can examine features such as social support and one’s involvement in the community. Social support can be understood as resources derived from relationships between persons that can facilitate different psychosocial outcomes [23]. Ample literature indicates that young people acquire social support from family, peers, significant others, and institutional agents such as teachers and social workers [23]. Moreover, social support is identified as a major correlate of mental health [24]. It can also enhance one’s functioning and protect them from adverse developmental outcomes because the effect of stressful life events or adversities on a person’s wellbeing is found to be affected by the amount of social support received [25].

Besides social support from close relationships such as family members, friends, and other adult support, Woolcock and Narayan [26] identified the significance of enhancing social capital such as social networks and recourses that stem from distant relationships such as acquaintances, teammates, and people with different backgrounds. In terms of precarious socio-political contexts, Barber [27] found that being integrated in social settings such as religion can significantly moderate the association between being involved in conflict and problematic behaviors amongst Palestinian youth. A literature review also indicated that the collapse of one’s social networks can lead to anomie, alienation, and poor social support, which has implications for mental disorders among those struggling amid unstable political situations [9]. 

Because young people are a crucial part of any community, the community factor has garnered attention in studies examining youth’s exposure to major political conflicts (e.g., [28]). Having fragmented ties to the community may predispose young people to mental distress in times of political conflict [29]. However, there are potential protective factors that can buffer against the adverse effects of various kinds of stressors in the community. For example, the perceived amount of empowerment an adolescent draws from his or her community can serve as an indicator of strengths-based community support [30]. A community level of youth empowerment can encourage young people to participate in community affairs and enhance their civic engagement, which can thus mitigate the sense of powerlessness among young people and promote positive developmental outcomes, especially amid an unstable socio-political environment [31,32]. Findings of an Italian study also indicated that it is important to provide adolescents with more opportunities to experience prosocial-oriented civic engagement in a community context in order to enhance the social wellbeing of Italian adolescents [10].

### 1.6. Conceptual Framework and Hypotheses

Adopting a socio-ecological perspective, a conceptual framework was developed to investigate the direct effects of stress arising from political life events, meaning in life, resilience, social support, associational social capital, perceived empowerment in the community, and perceived opportunities for civic engagement on youth mental health in Hong Kong. Furthermore, it also aimed to examine the moderating effects of meaning in life, resilience, social support, associational social capital, perceived empowerment in the community, and perceived opportunities for civic engagement in the relationship between adolescents’ stress due to political life events and their mental health.

**Figure 1:**
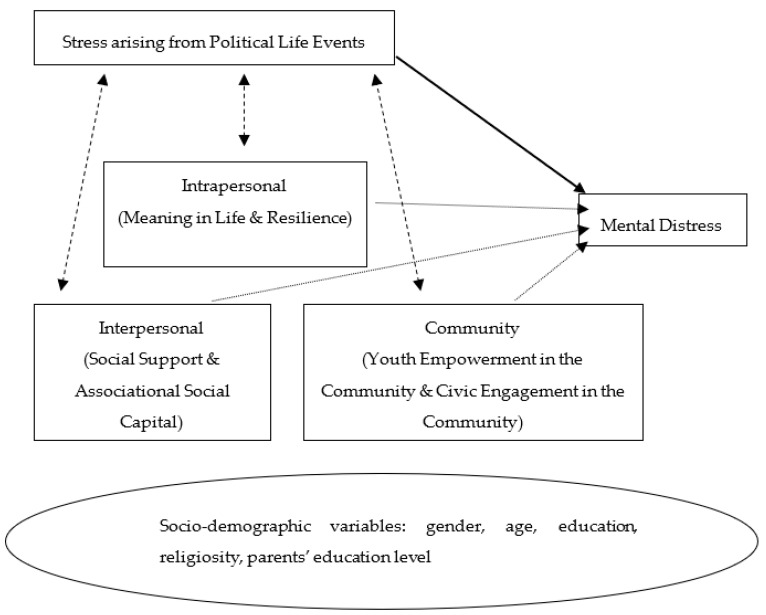
Conceptual Framework.

The basic premise of the study is that certain intrapersonal, interpersonal, and community protective factors may have moderating effects on the aforementioned relationship. This proposed framework can be illustrated in Figure 1:

As shown in the diagram, the solid arrow indicates the direct pathway between the predictor variable (adolescents’ stress arising from political life events) and the criterion variable (adolescents’ mental distress). The dotted arrow represents the direct influences of the moderating variables (intrapersonal, interpersonal, and community protective factors) on the criterion variable. The dashed arrow represents the ways in which the moderating factors interact with the predictor variable and affect the criterion variable. Based on this conceptual framework, the hypotheses of this study are derived as follows:

**Hypothesis** **1** **(H1).** *Adolescents who report a higher level of stress arising from political life events will report a higher level of mental distress.*

**Hypothesis** **2** **(H2).** *Adolescents who report a higher sense of meaningfulness in life will report a lower level of mental distress.*

**Hypothesis** **3** **(H3).** *Adolescents who report a higher level of resilience will report a lower level of mental distress.*

**Hypothesis** **4** **(H4).** *Adolescents who report a higher level of social support will report a lower level of mental distress.*

**Hypothesis** **5** **(H5).** *Adolescents who report a higher level of associational social capital will report a lower level of mental distress.*

**Hypothesis** **6** **(H6).** *Adolescents who perceive they have a higher level of youth empowerment in the community will report a lower level of mental distress.*

**Hypothesis** **7** **(H7).** *Adolescents who perceive they have more opportunities to be involved in civic engagement in the community will report a lower level of mental distress.*

**Hypothesis** **8** **(H8).** *Meaning in life, resilience, social support, associational socialcapital, perceived youth empowerment in the community, and civic engagement in the community have moderating effects on the relationship between adolescents’ stress arising from political life events and their mental distress.*

## 2. Materials and Methods

### 2.1. Sampling and Participants

We administered a cross-sectional quantitative survey targeting senior secondary students because middle and older adolescence is a crucial time for exploring meaning-making, personal identity, and self-reflection [4]. All adolescents are offered three years of compulsory senior secondary education under the present education system in Hong Kong. In the 2018/2019 school year, there was a total population of 155,727 senior secondary students [33]. Using G*Power, we set the effect size to 0.10, the alpha error probability to 0.05, the power for the F-test to 0.95, the number of the control variables and predictors to 12, and the minimum required sample size to 270 [34]. However, we proposed a target sample size of 1500 because, for a population over 150,000, a one-percent sample could be regarded as representative [35].

A stratified random sampling method was first adopted to select 10 schools to be representative of the study population. The five greater districts of Hong Kong (i.e., Hong Kong and Outlying Islands, Kowloon East, Kowloon West, the New Territories East, and the New Territories West) were treated as different stratums. Two coeducational second ary schools from each district were randomly selected. Invitation letters were sent to principals to solicit their support. If the responses of schools were not satisfactory due to the sensitive nature of this research and the possible disruptions caused by the pandemic, stratified purposive sampling would then be used.

Two rounds of random sampling were completed. A total of 20 coeducational secondary schools located in five greater districts were invited to participate in the survey. However, only one school responded to our invitation and agreed to join. Following the research plan, stratified purposive sampling was then adopted to select schools from different greater districts. The stratified purposive sampling was based on schools’ locations, religious affiliations, and medium of teaching language. Five coeducational secondary schools from different greater districts agreed to join. As a result, a total of six coeducational secondary schools located in five greater districts participated in the survey. The plan was to recruit more schools to join the survey; however, due to the outbreak of COVID-19 and the Education Bureau’s announcement of physical class suspension, the number of schools and students who responded to the research was less than expected. From October 2020 to March 2021, 1330 copies of the questionnaire were collected from senior secondary students (from 10th grade to 12th grade) of six coeducational secondary schools located in five greater districts. The response rate was 67.93%.

### 2.2. Measures of the Survey

Most of the measures used in this study were adapted from various scales in Western and Chinese literature. Details of the measures are as follows:

#### 2.2.1. Predictor Variable (Stress Arising from Political Life Events)

Based on two prior focus group sessions with adolescents to gather their experiences in political life events and their opinions of the content of the survey questionnaire, the authors developed a scale of 13 statements regarding stress arising from political life events. All 13 items were rated on a seven-point Likert-type scale from 1 (rarely/never) to 7 (always).

Sample items included “I experience stress because of differences in my political opinions with those of my family members or relatives”, “I experience stress because of differences in my political opinions with those of my friends or classmates”, and “I fear speaking up for my political stance under stress”. Total scores ranged from 13 to 91; a higher total scale score reflected a higher level of stress arising from political life events. A confirmatory factor analysis (CFA) was conducted to validate this scale using AMOS Software Version 25 (IBM SPSS Inc, Chicago, IL, USA). A variety of indices were examined to evaluate the goodness-of-fit, including the comparative fit index (CFI), the Tucker–Lewis index (TLI), and the root mean square error of approximation (RMSEA). The CFI and the TLI each ranged from 0 to 1, with 0 indicating no fit and 1 indicating a perfect fit [36]. The RMSEA ranged from 0 to 1, with values less than 0.05 considered an exact fit [36]. The goodness-of-fit indices for this measure were found to be satisfactory (CFI = 0.96, TLI = 0.99, RMSEA = 0.04). The Cronbach’s alpha coefficient was 0.927 in this study.

#### 2.2.2. Moderating/Protective Variables

Intrapersonal Factors—Meaning in Life: Adolescents’ sense of meaningfulness was assessed by The Meaning in Life Questionnaire—Presence of Meaning Subscale (MLQ-P), which was developed by Steger et al. [37] and translated by Chan [38].

The MLQ-P is a five-item scale including statements such as “I have a good sense of what makes my life meaningful”. Each item is rated on a 7-point Likert-type scale ranging from 1 (absolutely untrue) to 7 (absolutely true), thus the total score ranges from 5 to 35. The Cronbach’s alpha coefficient was 0.848.

Intrapersonal Factors—Resilience: Adolescents’ capacity for adapting to stressful events in healthy and effective ways was assessed by The Resilience Subscale of the Chinese Positive Youth Development Scale (CPYDS), which was developed by Shek et al. [39]. A sample item included “ I believe problems in life can be solved.”. Each item is rated on a 7-point Likert-type scale ranging from 1 (absolutely untrue) to 7 (absolutely true); thus, the total score ranges from 6 to 42. The Cronbach’s alpha coefficient was 0.849.

Interpersonal Factors—Social Support: Young people’s perceived social relationships and social support was assessed by The Social Provision Scale (SPS), which was developed by Cutrona and Russell [40] and translated into Chinese and validated by To et al. [41]. This is a 12-item scale containing statements such as “I have close relationships that make me feel good.”. Each item is rated on a 7-point Likert type scale (1 = strongly disagree; 7 = strongly agree) for total scores ranging from 12 to 84. The Cronbach’s alpha coefficient was 0.937.

Interpersonal Factors—Associational Social Capital: Young people’s perceived trust, solidarity, and social cohesion in associations or organizations was assessed by The Associational Social Capital Questionnaire (ASCQ), which was developed by Yuan and Ngai [42]. The ASCQ is a 10-item scale containing statements such as “People in this association can be trusted”. Each item is rated on a 5-point Likert-type scale (1 = strongly disagree; 5 = strongly agree) for total scores ranging from 10 to 50. The Cronbach’s alpha coefficient was 0.806.

Community Factor—Perceived Youth Empowerment in the Community: Young people’s perceived empowerment in their community was assessed by The Perceived Youth Empowerment Questionnaire (PYEQ), which was developed by Paxton et al. [43] and translated by To et al. [41]. The PYEQ is a 5-item scale containing statements such as “My neighborhood involves youth in important decisions.”. Each item is rated on a 7-point Likert-type scale (1 = strongly disagree; 7 = strongly agree) for total scores ranging from 7 to 35. The Cronbach’s alpha coefficient was 0.921.

Community Factor—Perceived Opportunities for Civic Engagement in the Community: The Behaviour Subscale of the Civic Engagement Scale (CES), developed by Doolittle and Faul [44], was adopted to measure the ways in which young people have opportunities to participate in the community in order to improve conditions for others or to help shape the community’s future. The CES is a 6-item scale containing statements such as “When working with others, I make positive changes in the community”. Each item is rated on a 7-point Likert-type scale (1 = rarely/never; 7 = always), for total scores ranging from 6 to 42. A CFA was conducted to validate this scale since the CES did not have a Chinese-language version. The goodness-of-fit indices for this scale were found to be satisfactory (CFI = 0.99, TLI = 0.99, RMSEA = 0.03). The Cronbach’s alpha coefficient was 0.925.

#### 2.2.3. Criterion Variable

Mental Health: The Chinese Hospital Anxiety and Depression Scale (HADS), developed by Zigmond and Snaith [45] and translated by Leung et al. [46], was used to measure the degree of depressive mood and anxiety symptoms. This scale comprises 14 items, 7 of which were designed to assess the degree of depressive mood symptoms and 7 to assess the degree of anxiety symptoms. Each item on the questionnaire is scored from 0 (e.g., not at all) to 3 (e.g., most of the time), which means a person can score between 0 and 21 for either anxiety or depression. Total scores range from 0 to 42; a higher total scale score reflects a higher level of mental distress. The Cronbach’s alpha coefficient was 0.807.

#### 2.2.4. Control Variables

Demographic Variables: The participants were asked to report some demographic data, including age, gender, educational level, religiosity, and parents’ education level. The influence of these demographic variables on the relationship patterns described in the moderation hypotheses was found in prior research (e.g., [14,47]), so the effects of these variables were considered controlled when conducting the regression analyses.

### 2.3. Data Collection

We obtained ethical approval from the Survey and Behavioural Research Ethics Committee of The Chinese University of Hong Kong prior to the study. We also obtained permission from the schools’ principals to conduct research with the students. Moreover, the informed written consents were obtained from the students and their parents prior to data collection. A self-administered anonymous survey was conducted in the classroom with the support of data collectors. Prior to administering the survey questionnaire, the data collectors briefed the participants on the aim of this study and assured participants about the confidentiality of information. They also emphasized that all data would be treated in the strictest confidence and no personal information would be disclosed to unauthorized parties. Then, the data collectors collected the questionnaires from participants immediately upon completion.

### 2.4. Data Analysis

Descriptive statistics were performed in the first phase of data analysis to explore the participants’ demographic characteristics. A Pearson’s correlation analysis was conducted to explore the correlations among all study variables, including demographic variables, predictors, and criterion variable (i.e., mental distress). A hierarchical regression analysis was conducted in the second phase of data analysis to investigate the relationship of mental distress with stress arising from political life events and intrapersonal, interpersonal, and community protective factors, as well as the moderating effects of those protective factors in the association between mental distress and stress due to political life events. If any of the demographic variables are statistically significantly related to mental distress, the significant demographic variable would first be entered in Step 1 of the regression model to control their influences. Stress arising from political life events would be entered in Step 2 of the regression model. Meaningfulness in life, resilience, social support, associational social capital, youth empowerment in the community, and civic engagement in the community would be entered in Step 3. Finally, an interaction term derived from stress due to political life events and each moderating/protective variable (e.g., stress due to political life events × meaningfulness in life) was created and entered in Step 4 of the regression model. The variables were mean-centered before the interaction term was created in order to avoid multicollinearity [48]. A significant moderating effect could be detected by plotting the regression equation at two levels (±1 *SD*) of each moderating variable. An alpha level of 0.05 would be used to indicate the statistical significance.

## 3. Results

### Participants

A total of 1330 adolescents were recruited for this study. Table 1 shows the demo-graphic characteristics of the participants. Of the 1330 participants, 53.9% were male and 46.1% were female. Of the total sample, 21.1% were aged 15 or below, 67.5% were aged 16–17, 11.2% were aged 18–19, and 0.2% were aged 20 or above. Among the participants, 74.1% reported having no religious beliefs. In terms of education level, 25.2% were in secondary four, 36.4% were in secondary five, and 38.4% were in secondary six. More than two thirds (69.9%) had a father whose highest education level was senior secondary school or above, and almost two thirds (65.7%) had a mother whose highest education level was senior secondary school or above.

Table 2 presents the means and standard deviations of all study variables and the zero-order correlations among the variables. The results offer tentative evidence that the predictor variable (stress arising from political life events), most of the presumed moderators (meaningfulness in life, resilience, social support, associational social capital, youth empowerment in the community, and civic engagement in the community), and the criterion variable (mental distress) are significantly correlated. Moreover, it is noteworthy that none of the demographic variables were found to be significantly correlated with mental distress. Hence, they were not controlled when conducting the regression analysis (see Table 2).

Table 3 reports the results of a hierarchical regression analysis for the relationship between mental distress, stress arising from political life events, and each presumed moderator. As indicated in Table 3, there is a positive association between stress arising from political life events and adolescents’ mental distress. Hence, Hypothesis 1 is supported. The results also show that higher levels of meaningfulness in life, resilience, social support, and youth empowerment in the community were significantly associated with lower levels of mental distress. Therefore, Hypotheses 2, 3, 4, and 6 were supported. However, civic engagement in the community was found to be positively associated with mental distress, which was contrary to Hypothesis 7. Furthermore, the results do not support Hypothesis 8, as no interaction effects could be found among the predictor variable and all presumed moderators (see Table 3).

## 4. Discussion

This study examined the effects of stress arising from political life events and intrapersonal, interpersonal, and community protective factors on the mental health of Hong Kong adolescents. While the impacts of stress due to political life events have rarely been investigated among adolescents in Hong Kong, the findings of the study contribute to mental health literature by suggesting that Hong Kong adolescents’ subjective experiences in political life events are an important factor for mental distress. Moreover, because there is little focus on the relationship between youth wellbeing and political circumstances in Chinese societies, this study also highlights different levels of protective factors on mental health in the context of precarious political situations. Specifically, this study explicated the effects of meaningfulness in life, resilience, social support, youth empowerment in the community, and civic engagement in the community on mental distress among Hong Kong adolescents. The finding that a higher level of stress arising from political life events is associated with a higher level of mental distress is consistent with the findings of previous studies on the impacts of political conflict on mental health [15]. Existing literature indicates that a sense of uncertainty is related to anxiety [49]. Possibly, during precarious political situations, adolescents worry about the outcomes for their participation in political events or for their expression of their political positions. These worries, in turn, arouse feelings of uncertainty, leading to a high level of anxiety. It is also noted in the literature that autonomy is an important factor for depression [50]. When adolescents are not able to freely express or act based on their political stances due to a fear of the outcomes, their sense of autonomy is compromised, which might evoke a sense of depression. Because stress arising from political life events differs from stress amid existential or survival issues, the way that it affects adolescent mental health deserves further exploration in future studies.

In this study, intrapersonal, interpersonal, and community protective factors (i.e., meaningfulness in life, resilience, social support, youth empowerment in the community, and civic engagement in the community) were significantly associated with mental health, as suggested by the existing literature [1,17,18,24,25,31,32,47,51]. However, none of these variables were found to be a significant moderator in the relationship between stress arising from political life events and mental health. Although social movements periodically occur in Hong Kong, the ELAB incident induced the most severe conflicts and violent events among all political life events, and dramatically changed many aspects of lives for people in Hong Kong. Additionally, the COVID-19 outbreak severely negatively affected the mental health of Hong Kong residents [52]. Potentially, due to the drastic changes in the political environment and the considerable impacts of COVID -19, inconsistent with our hypotheses, those presumed protective factors were not able to buffer the impacts of stress due to political life events on mental health. Studies examining the impacts of serious environmental or macro-social risks show the prevalence of mental distress and existential anxiety among young people with overwhelming traumatic experiences [2,3]; the stresses arising from fundamental concerns of how people and society would be profoundly changed by these events may not be easily dealt with or alleviated.

Moreover, as previous studies indicated the differences in emotional outcomes from different types of political conflicts [53], another possible explanation for the insignificant moderating effect is that stress arising from political life events for Hong Kong adolescents is so unique and challenging that its effect on mental health may not be mitigated by the formerly mentioned protective factors. For example, even though freedom of speech, the press, association, assembly, and demonstration are guaranteed in the Basic Law, there is heated debate on whether such kinds of freedom are threatened by the ELAB incident and the promulgation of national security law. The stress amid the ambivalence of whether or not and/or how to express one’s political views, as well as how political conflicts influence personal lives, might be a new and daunting challenge for Hong Kong adolescents. As the protective factors in our study fall in developmental domains, they could be limited in moderating the impact of stress arising from political life events on mental distress.

Although civic engagement in the community is insignificantly and negatively associated with mental distress at the bivariate level, it became positively associated with mental distress when controlling other protective factors (i.e., meaningfulness in life, resilience, social support, associational social capital, and youth empowerment in the community) in our study. The result is conflicting with previous study findings [10]. As indicated in Table 2, those with higher levels of meaning in life, resilience, social support, and empowerment in the community were more likely to have civic engagement, suggesting that there could be overlapping effects on mental health between civic engagement in the community and other protective factors. Hence, it is possible that when the effects of the above-mentioned protective factors are controlled, having a higher level of civic engagement in the community may induce burnout and frustration and, in turn, increase the level of psychological distress. The nature of civic engagement in the community and how it associates with other protective factors in explaining its relationship with mental health should be further explored in future studies. 

There are several limitations for this study. First, this is a cross-sectional study, which limits the causal inference for the results. Hence, the results should be interpreted with caution. Second, as the data were collected during the COVID -19 pandemic, the impacts of the outbreak were not counted in this study. Third, the responses could be biased due to social desirability since political stance was a sensitive topic during the data collection period. Fourth, recall bias was inevitable because this was a survey study based on self-reporting.

### Implications

As current mental health assessment and services in Hong Kong do not include a political dimension, our study findings suggest that assessing stress related to political life events is necessary in enhancing adolescents’ mental health. It is difficult to clearly articulate and fully understand the impact of precarious social-political climates on adolescent wellbeing without a political dimension. In addition, school and mental health social workers need to help adolescents talk about their experiences in precarious political situations. Proactive outreach services should be provided by non-governmental organizations to assist schools in the identification of adolescents who may be struggling with mental distress due to political tensions. The meaning of political life events and how political situations affect their mental health should also be explored and discussed in mental health programs.

Moreover, because our findings indicated that meaningfulness in life, resilience, social support, and youth empowerment in the community are inversely related to mental distress, interventions aimed to enhance the aforementioned factors should be taken into consideration in youth mental health services during or after political life events. Although these factors were not able to buffer the negative influence of stress arising from political life events on adolescent mental health, they could help compensate for the negative influence of stress due to political life events by exerting direct positive effects on adolescent mental health [54].

## 5. Conclusions

In conclusion, stress arising from political life events is a potentially important factor for mental distress among Hong Kong adolescents in precarious political situations. Several protective factors—such as higher levels of meaningfulness in life, resilience, social support, youth empowerment in the community, and a lower level of civic engagement in the community—were found to be related to a lower level of mental distress. The findings indicate the importance of addressing stress due to political life events among Hong Kong adolescents in precarious political situations. These findings can help us better understand how to respond to the recent political tensions and future stressful situations that may affect the wellbeing of youth. In addition, efforts aimed to address the aforementioned protective factors are potentially helpful for promoting mental health for Hong Kong adolescents. Further studies to understand how stress arising from political life events and certain protective factors contribute to better mental health are necessary.

## Figures and Tables

**Table 1:** Demographic Characteristics as a Percentage of the Sample (*n* = 1330).

Demographic Characteristic	%
Gender	
Male	53.9
Female	46.1
Age	
15 or Below	21.1
16–17	67.5
18–19	11.2
20 or Above	0.2
Religion	
No	74.1
Yes	25.9
Education Level	
Secondary 4	25.2
Secondary 5	36.4
Secondary 6	38.4
Father’s Education Level	
Primary School or Below	9.9
Junior Secondary School	20.1
Senior Secondary School	18.1
Matriculation	29.1
College/University	16.0
Postgraduate	6.7
Mother’s Education Level	
Primary School or Below	15.0
Junior Secondary School	19.3
Senior Secondary School	18.2
Matriculation	25.8
College/University	17.4
Postgraduate	4.3

**Table 2:** Correlations among demographic variables, criterion variables, predictor variables, and moderating variables with means and standard deviations (*n* = 1330).

	Var	1.	2.	3.	4.	5.	6.	7.	8.	9.	10.	11.	12.	13.	M (SD)
1.	Gen														
2.	Age	−0.061 *													16.41 (1.039)
3.	Rel	−0.024	−0.059 *												
4.	Edu	0.001	0.737 ***	−0.047											
5.	Fat’s Edu	−0.018	−0.048	0.135 ***	0.021										
6.	Mot’s Edu	0.017	−0.110 ***	0.128 ***	−0.075 **	0.648 ***									
7.	MH (DV)	−0.002	0.035	−0.021	0.043	−0.024	−0.037								16.601 (6.230)
8.	SPLE (IV)	0.005	0.015	0.076 **	0.093 ***	−0.020	−0.010	0.175 ***							48.932 (16.348)
9.	ML (MV)	−0.041	−0.009	0.123 ***	−0.010	0.051	0.035	−0.331 ***	0.004						21.847 (5.771)
10.	RE (MV)	−0.058	0.074 **	0.065 *	0.075 **	0.005	0.020	−0.400 ***	0.049	0.448 ***					28.192 (5.943)
11.	SS (MV)	0.078 **	0.022	0.037	0.056 *	0.027	0.068 *	−0.348 ***	0.120 ***	0.316 ***	0.481 ***				57.362 (13.261)
12.	ASC (MV)	−0.005	−0.036	0.076 **	−0.015	0.013	0.031	−0.111 ***	0.031	0.101 ***	0.153 ***	0.155 ***			36.222 (3.007)
13.	YEC (MV)	−0.037	−0.054	0.025	−0.108 ***	−0.031	0.000	−0.135 ***	0.138 ***	0.130 ***	0.226 ***	0.223 ***	0.121***		15.220 (5.933)
14.	CEC (MV)	0.123 ***	0.057 *	0.119 ***	0.074 **	0.090 **	0.068 *	−0.019	0.345 ***	0.152 ***	0.230 ***	0.228 ***	0.127 ***	0.377 ***	16.590 (8.001)

Note. Var = Varaiable; Gender (Gen) was dummied as 0 = female, 1 = male; Religion (Rel) was dummied as 0 = without religion, 1 = with religion. Education (Edu) = year of study; Father’s Education (Fat’s Edu) = year of study; Mother’s Education (Mot’s Edu) = year of study; MH = Mental Health; SPLE = Stress Arising from Political Life Events; ML = Meaningfulness in Life; RE = Resilience; SS = Social Support; ASC = Associational Social Capital; YEC = Youth Empowerment in Community; CEC = Civic Engagement in Community. * *p* < 0.05; ** *p* < 0.01; *** *p* ≤ 0.001.

**Table 3:** Hierarchical regression analysis predicting mental distress from stress arising from political life events and all levels of protective factors.

Criterion			
Mental Distress			
Predictor	∆R^2^	β	t
Step 1 Stress Arising from Political Life Events	0.031 ***	0.175	6.489 ***
Step 2 Protective Factors	0.233 ***		
Meaningfulness in Life		−0.160	−5.986 ***
Resilience		−0.235	−8.062 ***
Social Support		−0.204	−7.372 ***
Associational Social Capital		−0.034	−1.425
Youth Empowerment in Community		−0.064	−2.467 *
Civic Engagement in Community		0.065	2.368 *
Step 3 Interaction between Stress Arising from Political Life Events & Protective Factors	0.004		
Interaction between Stress from Political Life Events & Meaningfulness in Life		0.001	0.019
Interaction between Stress from Political Life Events & Resilience		−0.020	−0.669
Interaction between Stress from Political Life Events & Social Support		−0.014	−0.478
Interaction between Stress from Political Life Events & Associational Social Capital		0.028	1.153
Interaction between Stress from Political Life Events & Youth Empowerment in Community		0.048	1.826
Interaction between Stress from Political Life Events & Civic Engagement in Community		0.025	0.943
Total *R*^2^	0.268		

* *p* ≤ 0.05; *** *p* ≤ 0.001.

## Data Availability

The datasets generated during and/or analyzed during the current study are not publicly available due to datasets containing information that could compromise the privacy of research participants. The data that support the findings of this study are available from the corresponding author (S.-M.T.) upon reasonable request.
